# Ozone-Based Advanced Oxidation Processes for Primidone Removal in Water using Simulated Solar Radiation and TiO_2_ or WO_3_ as Photocatalyst

**DOI:** 10.3390/molecules24091728

**Published:** 2019-05-03

**Authors:** Manuel A. Figueredo, Eva M. Rodríguez, Manuel Checa, Fernando J. Beltran

**Affiliations:** Departamento de Ingeniería Química y Química Física. Instituto Universitario de Investigación del Agua, Cambio Climático y Sostenibilidad (IACYS). Universidad de Extremadura, 06006 Badajoz, Spain; manuelfigueredo@unex.es (M.A.F.); evarguez@unex.es (E.M.R.); mcheca@unex.es (M.C.)

**Keywords:** primidone, AOPs, photocatalytic ozonation, TiO_2_, WO_3_

## Abstract

In this work, primidone, a high persistent pharmacological drug typically found in urban wastewaters, was degraded by different ozone combined AOPs using TiO_2_ P25 and commercial WO_3_ as photocatalyst. The comparison of processes, kinetics, nature of transformation products, and ecotoxicity of treated water samples, as well as the influence of the water matrix (ultrapure water or a secondary effluent), is presented and discussed. In presence of ozone, primidone is rapidly eliminated, with hydroxyl radicals being the main species involved. TiO_2_ was the most active catalyst regardless of the water matrix and the type of solar (global or visible) radiation applied. The synergy between ozone and photocatalysis (photocatalytic ozonation) for TOC removal was more evident at low O_3_ doses. In spite of having a lower band gap than TiO_2_ P25, WO_3_ did not bring any beneficial effects compared to TiO_2_ P25 regarding PRM and TOC removal. Based on the transformation products identified during ozonation and photocatalytic ozonation of primidone (hydroxyprimidone, phenyl-ethyl-malonamide, and 5-ethyldihydropirimidine-4,6(1H,5H)-dione), a degradation pathway is proposed. The application of the different processes resulted in an environmentally safe effluent for *Daphnia magna*.

## 1. Introduction

Although present in waters at very low concentrations (µg L^−1^ or ng L^−1^), pharmaceuticals, personal care products, pesticides, etc., may cause adverse effects on aquatic organisms due to their toxicity and/or mutagenicity and/or endocrine disruptor character [1,2,3]. To date, most of these compounds known as contaminants of emerging concern (CEC) are not regulated, many of them being recalcitrant towards the classical operations applied in urban wastewater treatment plants (UWWTP). As a consequence, UWWTP effluents have been identified as the primary source of CEC in the environment [4]. Therefore, before the discharge or reuse of the effluents, the application of a tertiary treatment such as ozonation, activated carbon adsorption, membrane filtration, or advanced oxidation processes (AOPs) is highly recommended [5]. AOPs, based on the combination of different oxidants, catalysts and/or types of radiation, are able to completely remove CEC from water at ambient conditions through the generation of hydroxyl radicals (HO·) among other species [6]. The key point to evaluate the efficacy of an AOP is the ability to mineralize the organics present in the water matrix. Since the mineralization is usually not complete, transformation products (TP), in some cases more harmful than the parent compounds, can be formed [7,8]. 

Photocatalytic ozonation is a promising AOP that leads to the generation of hydroxyl radicals through multiple ways by the interaction of the different processes involved, i.e., ozonation, UV radiation and (photo)catalytic oxidation [1,9,10]. In this sense, literature already reports several works that show the efficiency of photocatalytic oxidation systems (with and without ozone) on the elimination of different pollutants from water and wastewater [10,11,12,13,14,15,16].

Titania, specifically P25 TiO_2_ from Degussa, is the most studied photocatalyst for water detoxification and disinfection due to its properties (low cost, no leaching, high photoactivity). However, apart from the problems related to its nanometric size to be separated from water, its high band gap (3.2 eV) makes useless the application of visible radiation. Current trends in photocatalytic research are focused on finding photocatalytic materials capable of being excited under visible light thus improving the efficiency of solar light as radiation source [17]. Tungsten trioxide, WO_3_, a relatively low cost and nontoxic semiconductor with a band gap of ~2.6 eV, presents photocatalytic activity under visible light [18,19]. Unfortunately, one of the main drawbacks of WO_3_ as photocatalyst is that, unlike TiO_2_, oxygen cannot be used as electron trapping agent since its redox potential is less positive than that of the WO_3_ conduction band [19,20,21], so the photogenerated electrons (e^−^) and positive holes (h^+^) recombine and the formation of reactive oxygen species takes no place. Ozone, with a higher redox potential, reacts with the e^−^ leading to the formation of the ozonide radical (O_3_·^−^) that further evolves to HO· [22,23]:

(1) $$e^{-}+O_{3}\;\overset{k=6.3\times 10^{10}M^{-1}s^{-1}}{\to }{\;O}_{3}^{-}\cdot \;\to \;\mathrm{HO}\cdot$$

**Figure 1:**
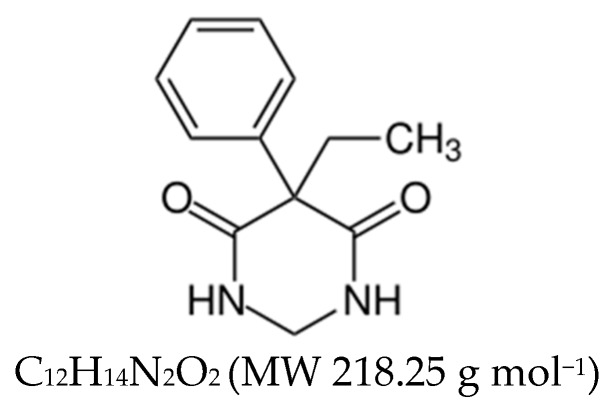
Chemical structure of primidone.

Primidone (5-ethyl-5-phenyl-1,3-diazinane-4,6-dione; see molecular structure in Figure 1), is an anticonvulsant of the barbiturate class used to treat movement disorders such as tremors, convulsion, Parkinson, etc., as well as migraines among other diseases. In humans, primidone (PRM) is partly eliminated unchanged via urinary excretion and partly metabolized by the liver to phenylethylmalonamide (2-ethyl-2-phenylpropanediamide, major metabolite) and phenobarbital (5-ethyl-5-phenyl-1,3-diazinane-2,4,6-trione) [24], whereas 2-hydroxyprimidone (5-ethyl-2-hydroxy-5-phenyl-1,3-diazinane-4,6-dione) has been proposed as a potential intermediate of both compounds [25].

PRM is usually found in UWWTP effluents. In fact, it was identified as one of the most recalcitrant compounds to conventional biological treatment (biological degradation < 20%) among 43 pharmaceuticals monitored in UWWTP [26]. Aminot et al. [27], from their study about the degradation and sorption of 53 pharmaceuticals present in UWWTP effluents discharged into simulated estuarine waters, identified PRM as one of the most stable with a persistence index of 100. Since PRM degradation rate at environmental conditions is low [28,29,30,31], its occurrence in different natural waters (groundwater, spring water, well water, and even drinking water catchment areas) has been reported [32,33].

Studies on the removal of PRM through the application of AOPs are scarce. Dong et al. [31] studied natural sunlight photodegradation of PRM added to ultrapure water ([PRM]_0_ 0.5 µg L^−1^) or present in two UWWTP secondary effluents (DOC~6.2 and 8 mg L^−1^; [PRM]_0_ 0.119–0.226 µg L^−1^). According to these authors, after five days, the amount of PRM degraded by direct photolysis was low (~5%), whereas ~35 and 88% of PRM was oxidized by the HO· generated from the photolysis of some organic/inorganic compounds present in the secondary effluents. Neamţu et al. [7] studied the degradation of PRM among other pharmaceuticals in ultrapure water, lake water, and a UWWTP effluent by UV-C, UV-C/H_2_O_2_ and UV-C/H_2_O_2_/Fe(II) ([PRM]_0_ 2 µM = 435 µg L^−1^ in all cases). A clear influence of the water matrix on pharmaceuticals removal was observed. Ternes et al. [34] reported a moderate reactivity of PRM toward ozone, and after applying 3 mg L^−1^ of O_3_ to a flocculated surface water spiked with 1 µg L^−1^ of PRM (pH 7.8, 23 °C), only 87% of the compound was converted. Real et al. also studied the application of ozone among other processes (UV-C and UV-/H_2_O_2_) to remove PRM from ultrapure and natural waters. When 2 mg L^−1^ of O_3_ was added to a groundwater (DOC 2.6 mg L^−1^, pH 7.5) or a surface water from a reservoir (DOC 6.7 mg L^−1^, pH 7.5) spiked with PRM 1 µM (~0.2 mg L^−1^), the conversion of PRM after 3 min was 83 and 35%, respectively. According to these authors, the rate constant of the reaction between ozone and PRM is low (k_O3-PRM_ = 1 M^−1^ s^−1^ regardless of the pH), whereas the rate constant of the reaction between PRM and HO· was 6.7 × 10^9^ M^−1^ s^−1^ [35]. More recently, the work of Checa et al. about PRM photocatalytic ozonation using a graphene oxide/titania catalyst in a Visible LED reactor has shown the ability of this composite to absorb visible light [36]. Identification of the first transformation products formed from PRM ozonation has not been reported. Liu et al., in their study about electron beam irradiation of PRM in aqueous solution, identified the formation of phenobarbital and hydroxyprimidone [37], whereas according to Sijak et al. [38], phenobarbital and hydroxyphenobarbital were formed during the photolysis of PRM under UV-C radiation.

The main objectives of the present work were to determine the efficiency of photocatalytic ozonation in water detoxification to remove PRM with two different catalysts, TiO_2_ P25 (Evonik) and WO_3_ (Sigma-Aldrich); two different types of solar radiation, global (UVA-Vis) and visible (Vis); in two different matrices, ultrapure water and a real secondary effluent from an UWWTP, identifying the main degradation byproducts and follow the ecotoxicity of treated samples to *Daphnia magna*.

## 2. Results

### 2.1. Degradation of PRM (5 mg L^−1^) in Ultrapure Water

A first experimental series was conducted in order to assess the efficacy of different AOPs on the removal of PRM ([PRM]_0_ 5 mg L^−1^; [TOC]_0_ 3.3 mg L^−1^; pH_0_~6, not buffered) in ultrapure water, using the global solar radiation (UV-Vis: λ > 300 nm) or only the visible fraction (Vis: λ > 390 nm)

#### 2.1.1. Photolysis, Ozonation and Photolytic Ozonation of PRM

**Figure 2:**
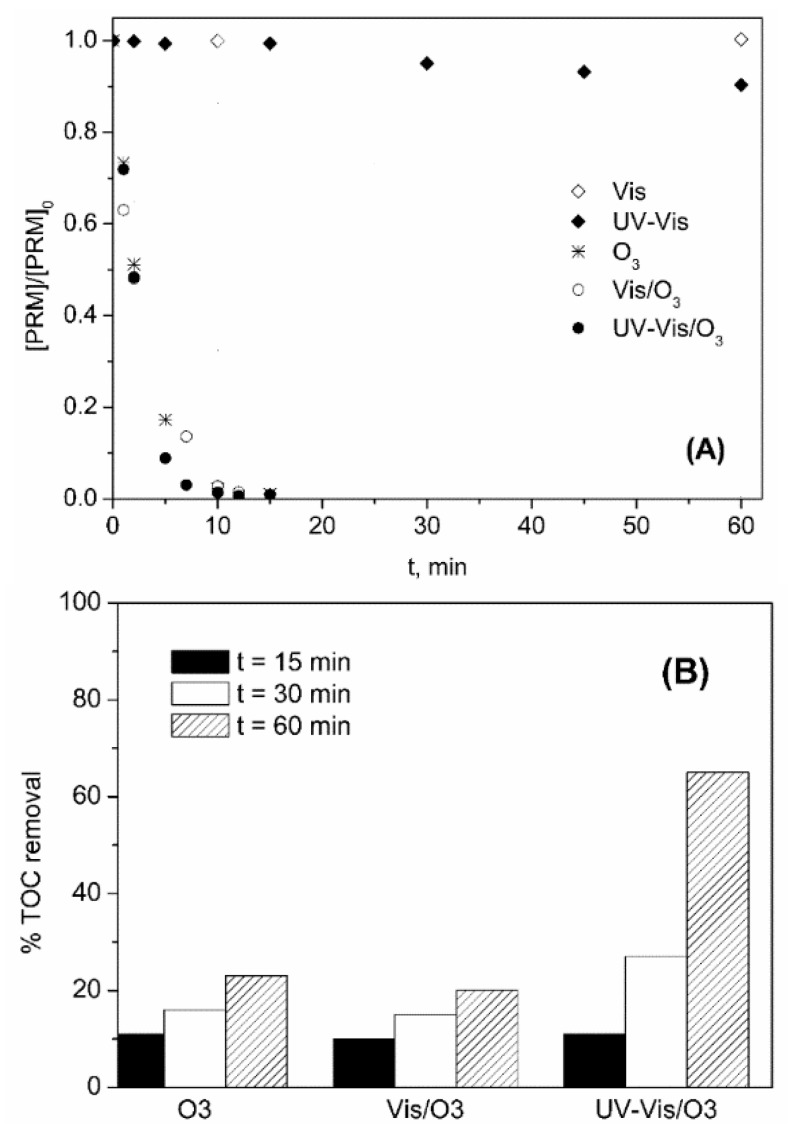
Variation with time of PRM normalized concentration (**A**) and percentage of TOC removed (**B**) during the application of different processes in ultrapure water. Experimental conditions: [PRM]_0_ = 5 mg L^−1^; [TOC]_0_ = 3.3 mg L^−1^; pH_0_~6 (natural pH); Q_m,O3_ = 3.3 mg min^−1^; T = 20–30 °C; I_Vis_ = 7.75 × 10^−5^ Einstein L^−1^ s^−1^; I_UV-Vis_ = 8.2 × 10^−5^ Einstein L^−1^ s^−1^.

**Figure 3:**
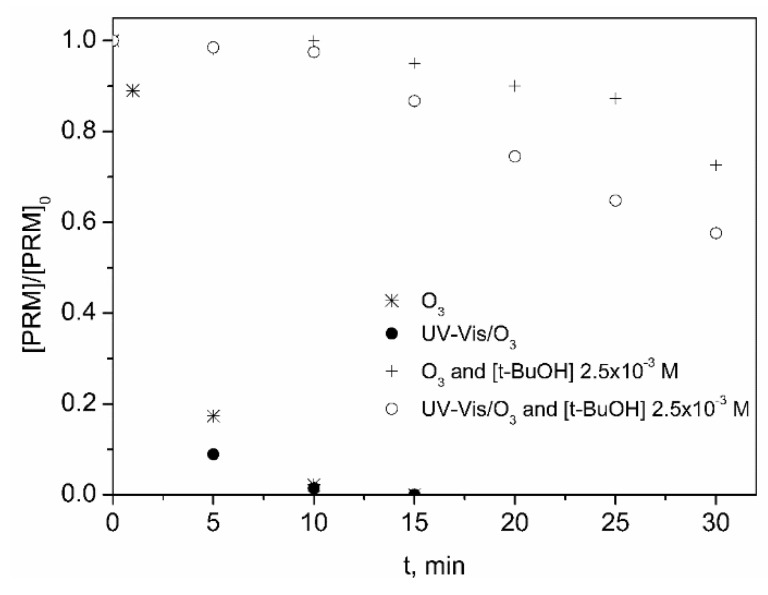
Influence of t-BuOH on the elimination of PRM by O_3_ and UV-Vis/O_3_ processes in ultrapure water. Experimental conditions: [PRM]_0_ = 5 mg L^−1^; [t-BuOH] = 2.5 × 10^−3^ M; pH_0_~6 (natural pH); Q_m,O3_ = 3.3 mg min^−1^; T = 20–30 °C; I_UVA-Vis_ = 8.2 × 10^−5^ Einstein L^−1^ s^−1^.

In Figure 2A the evolution with time of the normalized concentration of PRM corresponding to different oxidizing systems is shown. Also, Figure 2B depicts the variation of percentage of TOC removed with time for the same ozone-based treatments. As observed in Figure 2A, Vis radiation caused no photodegradation of PRM, whereas some photolysis occurred under UV-Vis (10% after 1 h). When ozone was fed, the degradation of PRM was fast, and total conversion was achieved in less than 20 min regardless of the presence of radiation. However, PRM elimination rate was higher under UV-Vis, the value of the apparent pseudo-first order rate constant being, k_Obs_, 0.32 and 0.43 min^−1^ (R^2^ > 0.98), for O_3_ and UV-Vis/O_3_, respectively. In any case, this high degradation rate was unexpected considering the low reactivity of PRM towards ozone (k_O3-PRM_ = 1.0 ± 0.1 M^−1^ s^−1^, obtained by competition kinetics [35]), and also that at the pH of the solution (pH_0_~6 that decreased to pH~5 after 60 min) the decomposition of O_3_ into HO· was not favored. Thus, the k_O3-PRM_ value was checked using a direct absolute method [39] (see Supplementary Information). After confirming the slow regime of ozone absorption [40], k_O3-PRM_ = 3.08 ± 0.14 M^−1^ s^−1^ was obtained, in agreement with Real et al. [35]. According to these results hydroxyl radicals most probably contribute to the removal of PRM by ozonation. To check this, some experiments of PRM ozonation ([PRM]_0_ 5 mg L^−1^ = 2.3 × 10^−5^ M) were performed adding tert-butanol (t-BuOH, [t-BuOH]_0_ 2.5 × 10^−3^ M) as HO· scavenger. At these conditions, taking into account the rate constant of the different reactions involved (k_O3-PRM_ = 3.08 M^−1^ s^−1^; this work; k_O3-tBUOH_ = 1.1 × 10^−3^ M^−1^ s^−1^ [41]; k_HO·-PRM_ = 6.7 × 10^9^ M^−1^ s^−1^ [35]; k_HO·-tBuOH_ = 6.2 × 10^8^ M^−1^ s^−1^ [42]), in case hydroxyl radicals are formed they will mainly react with the alcohol whereas O_3_ will react only with PRM. The influence of the presence of t-BuOH on the elimination of PRM by O_3_ and UV-Vis/O_3_ processes is shown in Figure 3.

As observed from Figure 3, there is no doubt, HO· is the main species responsible for the elimination of the contaminant by both systems, which means that PRM promotes somehow the decomposition of ozone into HO·, similarly to what has been observed for other amine-containing compounds [19,43,44].

With regard to mineralization, as it is seen in Figure 2B, TOC conversion during single ozonation was low (~20% after 1 h) and similar to that obtained with Vis/O_3_, which indicates that ozone does not decompose into HO· under visible light. On the contrary, TOC removal was clearly improved when ozone and UV-Vis were combined (65% after 1 h). Unlike Vis light, UV radiation promotes the decomposition of ozone through Reactions (2) and (3), the quantum yield in terms of HO· formation decreasing from 0.9 at 307 nm to 0.08 at 325 nm and 0.06 at 375 nm [45,46].

(2) $$O_{3}\;\overset{\mathrm{hv}}{\to }{\;O}_{2}+O\;{(}^{1}D)$$

(3) $$O{(}^{1}D)+H_{2}O\to 2\mathrm{HO}\cdot$$

The stationary concentration of dissolved ozone (C_O3d_) reached when O_3_ and UV-Vis/O_3_ processes were applied was ~3 × 10^−5^ M and 10^−5^ M, respectively. In both cases, C_O3d_ was negligible during the first 10 min (when PRM was still present, see Figure 2A) due to its decomposition into HO·. Once the compound was degraded this effect disappeared. Thus, the higher efficiency of the UV-Vis/O_3_ process in terms of mineralization seems to be directly related to the formation of HO· through Reactions (2) and (3), which highlights the effectiveness of this system in water treatment.

#### 2.1.2. TiO_2_ P25 As Photocatalyst

Different TiO_2_-based processes were applied ([TiO_2_] 0.25 g L^−1^) in order to assess the efficiency of photocatalytic oxidation and photocatalytic ozonation systems on the removal of PRM in ultrapure water ([PRM]_0_ 5 mg L^−1^; [TOC]_0_ 3.3 mg L^−1^), using solar UV-Vis and Vis radiation.

**Figure 4:**
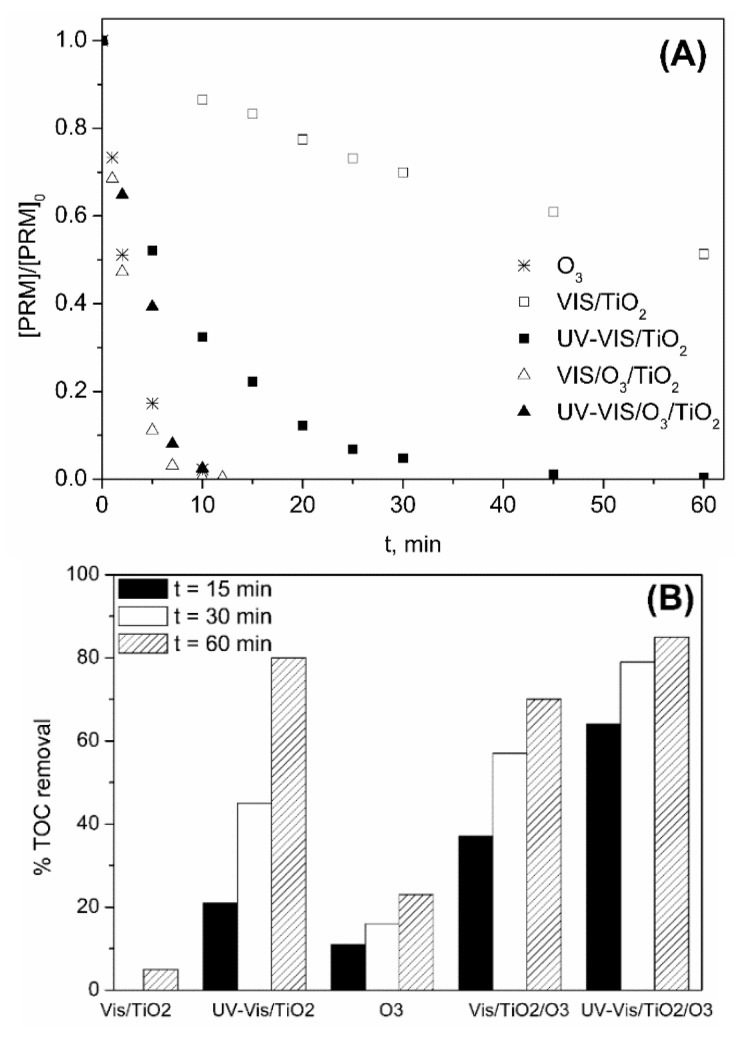
Variation with time of PRM normalized concentration (**A**) and percentage of TOC removed (**B**) during the application of different processes in ultrapure water. Experimental conditions: [PRM]_0_ = 5 mg L^−1^; [TOC]_0_ = 3.3 mg L^−1^; [TiO_2_] = 0.25 g L^−1^; pH_0_~6 (natural pH); Q_m,O3_ = 3.3 mg min^−1^; T = 20–30 °C; I_Vis_ = 7.75 × 10^−5^ Einstein L^−1^ s^−1^; I_UV-Vis_ = 8.2 × 10^−5^ Einstein L^−1^ s^−1^.

In Figure 4, the changes with time of PRM concentration and TOC removal percentage from different photocatalytic oxidation and ozonation processes are shown. For comparison purposes, data corresponding to single ozonation are also included. In all cases, adsorption of PRM onto TiO_2_ was negligible.

As observed in Figure 4A, removal of PRM was fast and took place at a similar rate for all the ozone-based processes. In absence of ozone (photocatalytic oxidation), PRM degradation rate was clearly higher under UV-Vis compared to Vis (k_Obs_ 0.093 and 0.012 min^−1^ for UV-Vis/TiO_2_ and Vis/TiO_2_, respectively; R^2^ > 0.99). According to the band gap of TiO_2_ P25 (3.2 and 3.0 eV for anatase and rutile, respectively [47]), this catalyst could be excited by radiation of wavelength up to 400 nm. Hence, from the results obtained some contribution of wavelengths in the frontier between UV-A and visible light (λ < 390 nm cut-off filter) for the Vis/TiO_2_ system is observed.

**Figure 5:**
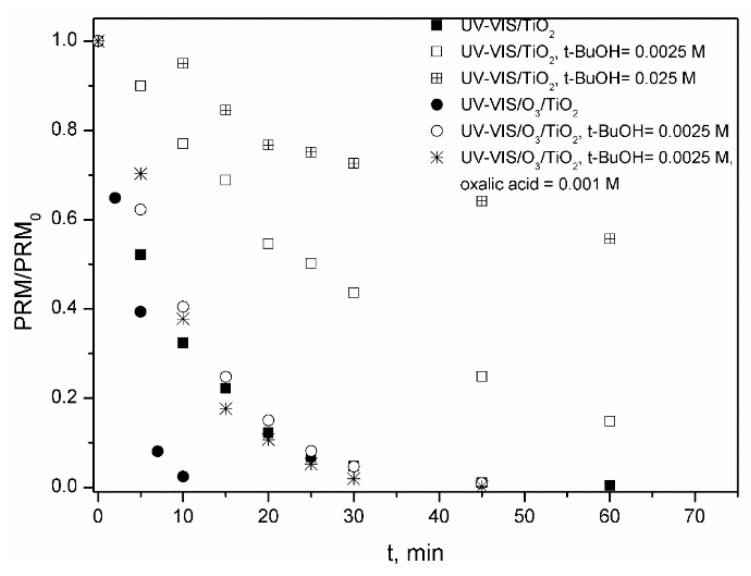
PRM removal in the presence and absence of t-butanol in UV-Vis/TiO_2_ and UV-Vis/TiO_2_/O_3_ systems and in the presence oxalic acid in UV-Vis/TiO_2_/O_3_ system. Experimental conditions: Ultrapure water; [PRM]_0_ = 5 mg L^−1^; pH_0_~6 (natural pH); Q_m,O3_ = 3.3 mg min^−1^; T = 20–30 °C; I_UV-Vis_ = 8.2 × 10^−5^ Einstein L^−1^ s^−1^.

The involvement of HO radicals in bulk water on PRM degradation by TiO_2_ photocatalysis (in the presence and absence of ozone) was determined using t-BuOH as a HO· scavenger [35]. As shown in Figure 5, the presence of t-butanol reduced PRM removal rate, which clearly indicates an important participation of bulk water HO radicals. However, in photocatalytic oxidation processes, oxidizing holes (formed in the valence band) and electrons (from the conduction band) can also participate in the oxidation. According to the well-known TiO_2_ photocatalytic oxidation process, formed holes can react, directly or through adsorbed hydroxyl radicals formed from their reaction with hydroxyl groups or adsorbed water, with the adsorbed matter. In this work, however, PRM was not adsorbed on the catalyst so that hole contribution could be neglected [48]. In any case, some experiments of photocatalytic ozonation were carried out in the presence of oxalic acid, as hole scavenger [49]. As shown in Figure 5, PRM removal rate was independent on the presence or absence of this scavenger which supports the previous conclusion. Note, however, that in the absence of ozone, oxalic acid was not used because, as Andreozzi et al. reported [50], this acid also reacts with HO radicals to form the ·COOH radical:

(4) $$\mathrm{HOOC}-\mathrm{COOH}+\mathrm{HO}\cdot \;\to {\;\mathrm{CO}}_{2}+\cdot \mathrm{COOH}$$

According to reaction (4), if oxalic acid is used in ozone-free photocatalytic oxidation, the results could induce some misunderstanding. Nonetheless, for photocatalytic oxidation, application of a high concentration of t-butanol, gives rise to an increase of PRM removal rate inhibition (see Figure 5), which confirms the main participation of HO radicals in the process. When ozone is present this problem does not take place since ·COOH radical reacts with ozone to regenerate the hydroxyl radical [50]:

(5) $$\cdot \mathrm{COOH}+O_{3}\to \mathrm{HO}\cdot \;+{\;\mathrm{CO}}_{2}+O_{2}$$

and according to Reactions (4) and (5) the presence of oxalic acid would not have any influence on the net formation rate of HO radicals.

Electrons, on the other hand, do affect PRM oxidation rate. In the presence of ozone, reaction (1) develops, yielding the ozonide ion radical which eventually gives the HO radical. In fact, this is the reaction that explains the synergism between ozonation and photocatalytic oxidation. In the absence of ozone, oxygen traps electrons to yield the superoxide ion radical:

(6) $$e^{-}+O_{2}\;\to {\;O}_{2}^{-}\cdot$$

Recombination of the protonated form of this radical (HO_2_ radical) yields hydrogen peroxide [51]:

(7) $$2{\mathrm{HO}}_{2}^{}\cdot +H_{2}O\to H_{2}O_{2}^{}+O_{2}+{\mathrm{OH}}^{-}$$

Notice that reaction (6) also develops when ozone is present, in UV-Vis/O_3_/TiO_2_ process, but here ozone also reacts with the superoxide ion radical to form more ozonide ion radicals:

(8) $$O_{2}^{-}\cdot +O_{3}\;\to {\;O}_{3}^{-}\cdot +O_{2}\;$$

According to these reactions, in both UV-VIS/O_3_/TiO_2_ and UV-VIS/TiO_2_ processes, hydrogen peroxide (H_2_O_2_) is formed. This oxidant was identified in this work and its concentration followed with time, as shown in Figure S3. It can be observed, from Figure S3, that hydrogen peroxide concentration increases with time until a maximum value and then it decreases likely as a consequence of reactions with electrons and hydroxyl radicals. Then, as oxidant, hydrogen peroxide also traps electrons to form hydroxyl radicals in bulk water [52]:

(9) $$e^{-}+H_{2}O_{2}\;\to \;\mathrm{HO}\cdot +{\mathrm{OH}}^{-}\;$$

Reactions (1) and (6)–(9) show how electrons can participate in PRM removal both in UV-VIS/O_3_/TiO_2_ and UV-VIS/TiO_2_ systems. As a resume, it can be said that hydroxyl radicals in bulk water, formed through different routes, are the main responsible species of PRM removal in UV-VIS/O_3_/TiO_2_ and UV-VIS/TiO_2_ systems.

Regarding TOC removal, as shown in Figure 4B, the highest mineralization rate was observed for UV-VIS/TiO_2_/O_3_ system being ~65% the TOC removed in 15 min, a value higher than the sum of TOC % removed by O_3_ (~10%) and UVA-VIS/TiO_2_ (~20%). Thus, the benefit of combining UV-Vis solar radiation, TiO_2_ and low doses of O_3_ in terms of PRM mineralization is clear. At longer treatment times (which imply higher O_3_ doses) the benefits are not so evident. Thus, after 1 h, % of TOC removed by UV-VIS/TiO_2_ and UV-VIS/TiO_2_/O_3_ was practically the same.

#### 2.1.3. WO_3_ As Photocatalyst

**Figure 6:**
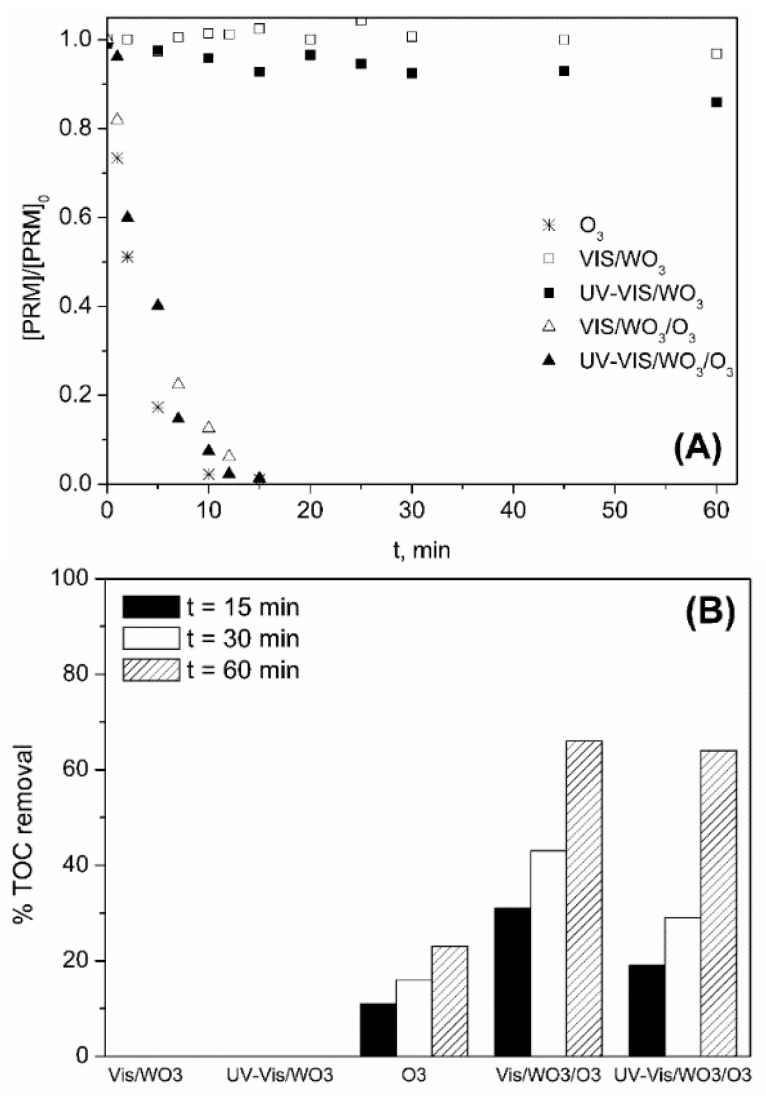
Variation with time of PRM normalized concentration (**A**) and percentage of TOC removed (**B**) during the application of different processes in ultrapure water. Experimental conditions: [PRM]_0_ = 5 mg L^−1^; [TOC]_0_ = 3.3 mg L^−1^; [WO_3_] = 0.25 g L^−1^; pH_0_~6 (natural pH); Q_m,O3_ = 3.3 mg min^−1^; T = 20–30 °C; I_Vis_ = 7.75 × 10^−5^ Einstein L^−1^ s^−1^; I_UV-Vis_ = 8.2 × 10^−5^ Einstein L^−1^ s^−1^.

Different WO_3_-based processes were also applied ([WO_3_] 0.25 g L^−1^) to assess the efficiency of photocatalytic oxidation and photocatalytic ozonation systems on the removal of PRM in ultrapure water ([PRM]_0_ 5 mg L^−1^; [TOC]_0_ 3.3 mg L^−1^), using UV-Vis and Vis solar radiation. Figure 6 shows the experimental results and data corresponding to single ozonation, also included for comparative purposes. Adsorption of PRM onto WO_3_ was negligible.

As observed in Figure 6A, removal of PRM was fast and took place at a similar rate for all the ozone-based processes. In absence of ozone (photocatalytic oxidation), unlike TiO_2_, PRM was not degraded regardless of the type of radiation used. These results are directly related to the redox potential of the WO_3_ conduction band, more positive than that of oxygen [16,17]. This implies that oxygen has not enough oxidizing power to trap the electrons jumped to WO_3_ conduction band so the generation of HO· does not take place. Contrary to oxygen, ozone has a more positive redox potential than WO_3_ conduction band and can act as electron trapping agent [20,21], which explains the behavior of the different systems in terms of TOC mineralization shown in Figure 6B. According to this figure, after 15 min the amount of TOC removed by O_3_, VIS/WO_3_/O_3_, and UV-VIS/WO_3_/O_3_ was ~10, 30, and 20%, respectively. Therefore, some positive effect of the presence of WO_3_ is deduced. The initial TOC conversion rate resulted to be higher when Vis instead of UV-VIS radiation was used which is related to the effect of the type of radiation on O_3_ photodecomposition, discussed in Section 2.1.1. Thus, under UV-VIS radiation, dissolved ozone photolyzes (Reactions (2)–(3)) and its concentration in water, together with its electron trapping role, diminishes. 

Finally, comparison of radiation/TiO_2_/O_3_ and radiation/WO_3_/O_3_ systems (Figure 4B and Figure 6B, respectively), leads to the conclusion that the use of WO_3_ does not bring any beneficial effects compared to TiO_2_ P25.

#### 2.1.4. PRM Transformation Products

Single ozonation (O_3_) and photocatalytic ozonation (UV-VIS/TiO_2_/O_3_) processes were selected to investigate the nature of PRM transformation products ([PRM]_0_ 5 mg L^−1^) and to elucidate a possible degradation route. As indicated in previous sections, for both processes a fast PRM degradation was observed during the first minutes of reaction (see Figure 4A) whereas the highest TOC mineralization was reached in photocatalytic ozonation (see Figure 4B).

**Figure 7:**
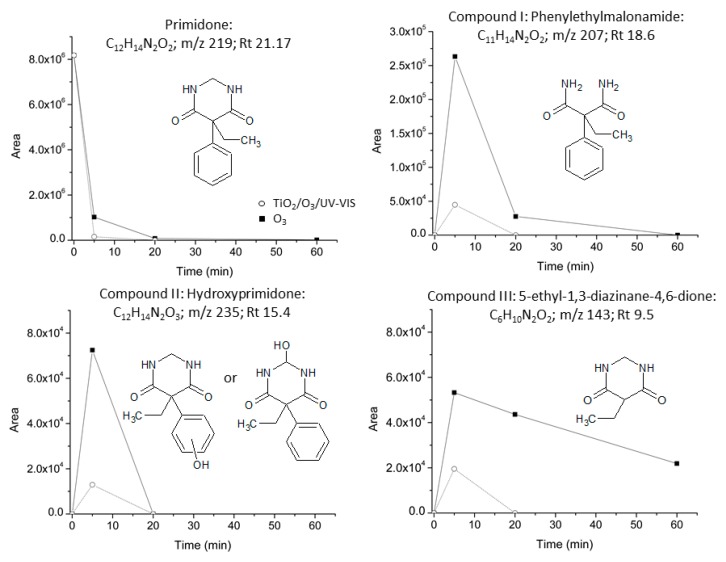
Evolution with time of the area of PRM and the intermediates detected by LC-QTOF-MS/MS during the application of O_3_ and UV-VIS/TiO_2_/O_3_ systems in ultrapure water. Experimental conditions as in Figure 4.

By LC-QTOF-MS analysis, three main initial intermediates were identified: Phenylethylmalonamide (PEMA, *m*/*z* 207, compound I), monohydroxylated primidone (*m*/*z* 235, compound II), and a compound that has been tentatively assigned to 5-ethyl-hexahydropyrimidine-4,6-dione (*m*/*z* 143, compound III). The evolution with reaction time of their signal intensity together with that of PRM during the application of O_3_ and UV-VIS/TiO_2_/O_3_ processes is shown in Figure 7. 

The initial intermediates formed were the same regardless of the system applied, which is a logical result since HO· radical is the main species involved in both oxidation processes, as discussed in previous sections. However, although PRM conversion rate was practically the same, the concentration of the intermediates in solution and the time needed for their total removal was higher when single ozonation instead of photocatalytic ozonation was applied, more markedly in the case of compound (III). These results are in agreement with the greater ability of the combined system to generate HO·.

There is no doubt that PEMA (I) is formed through the cleavage of the pyrimidine ring. However, its formation was not detected neither by Liu et al. or by Sijak et al. in their studies about the degradation of PRM by electron beam irradiation and UV-C photolysis, respectively [37,38]. On the contrary, these authors identified the formation of phenobarbital as one of the main intermediates, a compound that was not detected in the present work.

In case phenobarbital is formed from the oxidation of PRM by the application of O_3_ and UV-VIS/TiO_2_/O_3_ processes, its reactivity would be so high that it would be quickly transformed into other compounds which would explain why it was not detected in this work. Nonetheless, Cao et al. [53], in their study about the photocatalytic oxidation of phenobarbital (*m*/*z* 233) using TiO_2_ P25 and UV-A radiation (365 nm), identified the initial formation of hydroxyphenobarbital (*m*/*z* 249), PEMA and a compound with *m*/*z* 164 as the main intermediates with concentrations that further decreased slowly. In our case, formation of PEMA at least in part through the degradation of phenobarbital could be a possible way of PRM oxidation reaction mechanism. This possible route of PEMA formation would be minor since neither phenobarbital, hydroxyphenobarbital, nor a compound of *m*/*z* 164, were detected.

The intermediate with *m*/*z* 235 (compound II) was also identified by Liu et al. [37] and assigned to a hydroxylated primidone formed by the electrophilic addition of HO· to PRM aromatic ring. However, PRM hydroxylation could also take place at the C2 position leading to 2-hydroxyprimidone (5-ethyl-2-hydroxy-5-phenyl-1,3-diazinane-4,6-dione), compound that has been proposed as a potential intermediate of the formation of PEMA and phenobarbital during the metabolic degradation of PRM [25].

**Figure 8:**
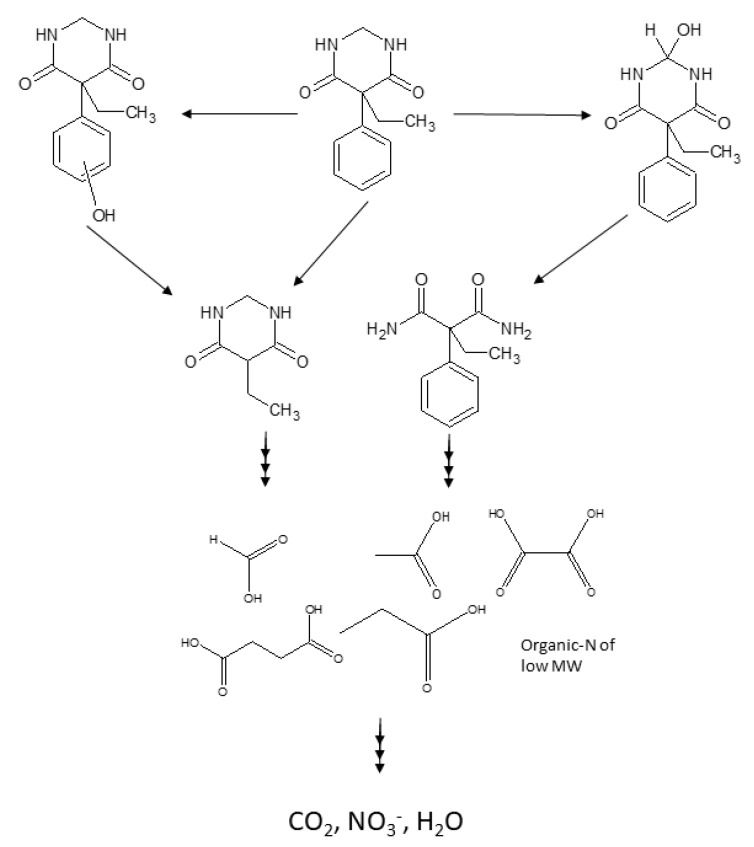
Tentative pathway of PRM degradation by O_3_ and UV-Vis/TiO_2_/O_3_.

According to the intermediates detected, a possible route for PRM degradation is tentatively proposed and shown in Figure 8.

In any case, these first intermediates are further oxidized to carboxylic acids such as acetic, propionic, formic, succinic, and oxalic acids, also detected in this work, that eventually evolve to CO_2_ and H_2_O.

Concerning inorganic anions and taking into account the presence of N on PRM structure, the evolution of the concentration of nitrate and nitrite ions was measured. Thus, the amount of N converted to NO_3_^−^ after 60 min was ~50% for the UV-Vis/TiO_2_/O_3_ system (TOC removal ≈ 80%, see Figure 4B), and negligible in the case of single ozonation (TOC removal ~ 25%), in all cases the concentration of NO_2_^−^ being below the LOD (0.18 mg L^−1^). The possible adsorption of NO_3_^−^ onto the catalyst was tested by putting in contact 10 mg L^−1^ of NO_3_^−^ in ultrapure water with 0.25 g L^−1^ of TiO_2_ P25 in the dark. After 1 h, no adsorption of NO_3_^−^ was observed. Similarly, a solution containing 10 mg L^−1^ of NO_3_^−^ was treated by UV-Vis/TiO_2_ and after 1 h the amount of nitrate remained the same, as expected. Therefore, UV-VIS/TiO_2_/O_3_ system results to be a much more efficient method than ozonation for the total oxidation of organic C and N, in all cases the mineralization degree of organic N being clearly lower than that of organic C.

#### 2.1.5. Ecotoxicity

Treatment of contaminants with AOPs in some cases may lead to intermediates of higher toxicity than the parent compounds [54]. In this work, *D. magna* test was used to check this. Ecotoxicity of PRM solutions before and after being treated ([PRM]_0_ 5 mg L^−1^; systems selected: UV-VIS, O_3_, UV-VIS/O_3_, UV-VIS/TiO_2_, UV-VIS/TiO_2_/O_3_, and VIS/WO_3_/O_3_; reaction time 60 min) was determined in terms of *D. magna* immobilization after 48 h. Before the assay, all the samples were diluted 1/8 with the culture medium in order to obtain concentrations of PRM and its intermediates that although still high ([PRM]_0,diluted_~0.6 mg L^−1^), were closer to that found in real UWWTP. Regardless of the treatment applied, the immobilization after 48 h was less than 10%. Therefore, it can be concluded that at the conditions tested the samples did not present acute toxicity to *D. magna*.

### 2.2. Degradation of PRM in A Secondary Effluent

**Figure 9:**
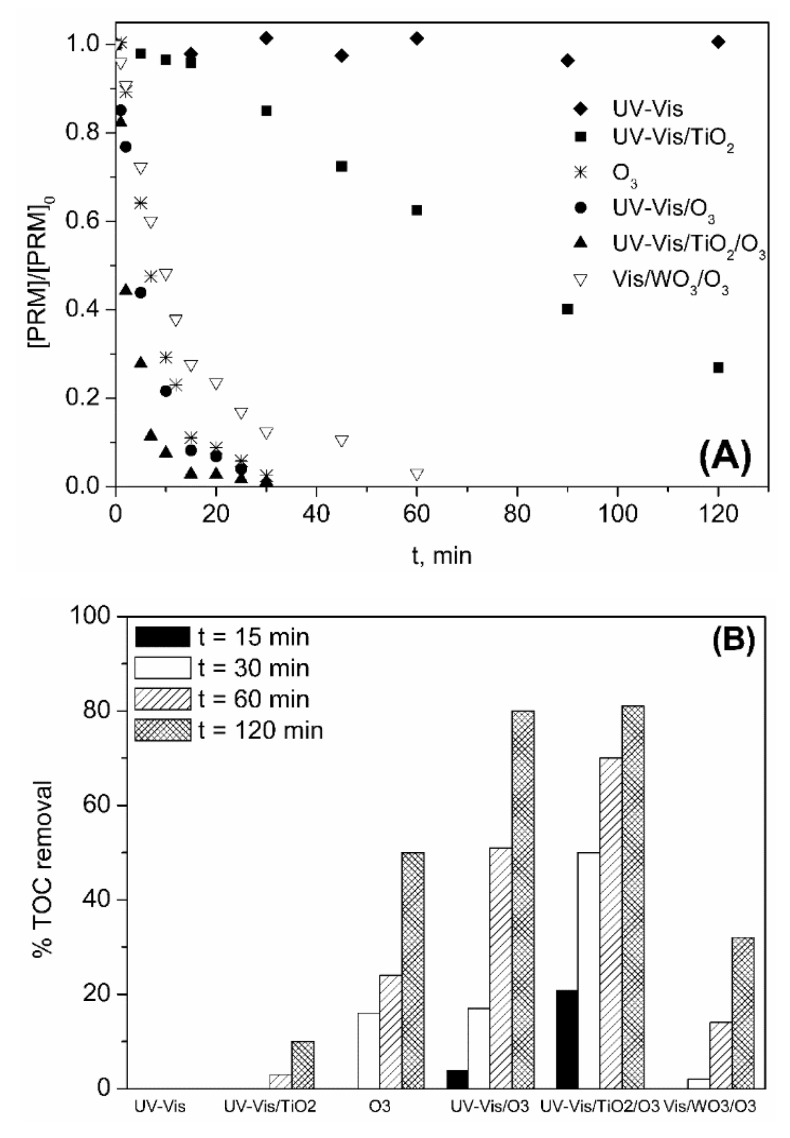
Variation with time of PRM normalized concentration (**A**) and percentage of TOC removed (**B**) during the application of different processes in SE wastewater. Experimental conditions (see also Table 1): [PRM]_0_ = 5 mg L^−1^; [TOC]_0-PRM_ 3.3 mg L^−1^; [TOC]_0-SE_ = 11 mg L^−1^; [TiO_2_] = 0.25 g L^−1^; [WO_3_] = 0.25 g L^−1^; pH_0_ 8.2; Q_m,O3_ = 3.3 mg min^−1^; T = 20–37 °C; I_Vis_ = 7.75 × 10^−5^ Einstein L^−1^ s^−1^; I_UV-Vis_ = 8.2 × 10^−5^ Einstein L^−1^ s^−1^.

To test the influence of the water matrix, in a last experimental series a secondary effluent (SE) collected from the UWWTP of Badajoz (Spain) was spiked with PRM ([PRM]_0_ 5 mg L^−1^) and treated for 2 h by different AOPs at the same experimental conditions as in ultrapure water. Main characteristics of SE after being filtered (Whatman paper, Grade 1) are compiled in Table 1. The processes selected were: UV-VIS, O_3_, UV-VIS/O_3_, UV-VIS/TiO_2_, UV-VIS/O_3_/TiO_2_, and VIS/O_3_/WO_3_. The results for both PRM and TOC removal in SE are presented in Figure 9.

TOC values before/after the adsorption period (30 min in the dark; not shown) were practically the same, which means that the adsorption of organics onto the surface of the catalyst was negligible. Regarding the pH, after 2 h, it increased from 8.2 to 8.5–9 (depending on the system applied). 

The results obtained in terms of PRM elimination correlated well with those obtained in ultrapure water. Thus, PRM degradation rate was fast for all ozone-based processes with PRM concentration under the LOD (100 µg L^−1^) in less than 60 min, the efficiency following the order: UV-VIS/TiO_2_/O_3_ (k_Obs_ = 0.254 min^−1^) > UV-VIS/O_3_ (k_Obs_ = 0.140 min^−1^) > O_3_ (k_Obs_ = 0.122 min^−1^) > VIS/WO_3_/O_3_ (k_Obs_ = 0.073 min^−1^). Since PRM must compete for the HO· with other organics and inorganics (bicarbonates/carbonates among them) present in SE, k_Obs_ values were lower than those obtained in ultrapure water (see values of k_Obs_ in ultrapure water and SE compiled in Table 2), though this competition effect was not very strong (k_Obs(SE)_/k_Obs(ultrapure)_ > 0.3). Aspects that smooth the competition effect are: (i) the role of PRM in O_3_ decomposition; (ii) the higher pH of the SE that also favors O_3_ decomposition; and (iii) the high reactivity of PRM towards HO· (k_HO·-PRM_ = 6.7 × 10^9^ M^−1^ s^−1^).

The effect of the matrix was more relevant in the case of the UV-VIS/TiO_2_ system. Thus, during the first 15 min, PRM was practically not degraded, which means that HO· generated during this period from the photoexcitation of TiO_2_ are consumed in other reactions. As shown in Table 1, SE had 14.16 mg L^−1^ of TOC, being the presence of this organic matter likely the reason of the lower PRM degradation rate. Once these organics are degraded, after 15 min, PRM started to be oxidized, being k_Obs(SE)_/k_Obs(ultrapure)_~0.1 (see Table 2). Also, carbonate/bicarbonate content (240 mgL^−1^ alkalinity as CaCO_3_) can also contribute to slow PRM oxidation rate.

Degradation of PRM in SE under UV-VIS was not observed. Therefore, neither direct nor indirect PRM photolysis took place. Thus, in case reactive oxygen species (HO·, singlet oxygen, superoxide radicals, etc.) are generated through the photolysis of compounds present in SE, at the conditions tested their contribution to PRM degradation was minimal.

Regarding TOC removal, as shown in Figure 9B the highest mineralization rate was observed for UV-Vis/TiO_2_/O_3_ system being ~20% of the TOC removed in 15 min, whereas no mineralization was attained at this time for O_3_ or UV-VIS/TiO_2_ and was < 5% for UV-VIS/O_3_. As in ultrapure water, the benefit of combining UV-VIS solar radiation, TiO_2_ and low doses of O_3_ in terms of PRM mineralization is clear. At much longer treatment the benefits are less evident. Thus, after 1 h TOC removed by UV-VIS/O_3_ and UV-VIS/TiO_2_/O_3_ was ~50% and 70%, respectively, and practically the same after 2 h (~80%).

Another aspect to highlight is the lower efficiency of the VIS/WO_3_/O_3_ system compared to O_3_ in terms of mineralization, contrary to what was observed in ultrapure water (see Figure 6B). The explanation is likely due to the lower concentration of dissolved ozone in the experiments performed in SE. In this sense, whereas for the ozonation of PRM in ultrapure water a stationary value of C_O3d_~5 × 10^−5^ M was reached, it resulted to be ten times lower in SE attributable, at least in part, to the higher pH of the medium. Therefore, the ozone available to act as e^−^ trapping agent and generate HO· (reaction (1)) was practically negligible, what makes the Vis/WO_3_/O_3_ system not appropriate in wastewater treatment.

As previously mentioned, the mineralization achieved by the application of UV-VIS/TiO_2_ system was very low (~10% after 2 h), in agreement with the low degradation rate of PRM observed. Although TOC removal rate in ultrapure and SE cannot be compared (due to different TOC_0_ values), no doubt that among the AOPs tested the SE matrix exerts the strongest negative influence on the efficiency of UV-VIS/TiO_2_ system. One of the possible reasons would be the aggregation and even deposition of the catalyst particles due to alkalinity content as demonstrated by different authors [55,56,57]. If this is the case, the good results obtained for the UV-VIS/TiO_2_/O_3_ system could be an indicator that O_3_ prevents the aggregation of TiO_2_ P25 particles, aspect that will be investigated in future works.

Finally, to bring conditions closer to the real ones, SE was spiked with 100 µg L^−1^ of PRM and treated by VIS/WO_3_/O_3_ or UV-VIS/O_3_/TiO_2_ systems. The time needed to reduce PRM under the LOD of the method (5 µg L^−1^) was ~10 min in both cases (k_Obs_ 0.09 and 0.11 min^−1^ for VIS/WO_3_/O_3_ and UV-VIS/O_3_/TiO_2_, respectively).

## 3. Materials and Methods

### 3.1. Materials

Primidone (analytical grade) was obtained from Fluka, commercial WO_3_ was from Sigma-Aldrich (S_BET_ 8.3 m^2^ g^−1^, band gap 2.61 eV [58]) and TiO_2_ P25 Aeroxide^®^ from Evonik Industries (Essen, Germany) (S_BET_ 50 m^2^ g^−1^, anatase to rutile ratio 5.3 ± 0.28, band gap 3.2 and 3.0 eV for anatase and rutile, respectively [47]). Other reagents were at least of analytical grade and used as received. Ultrapure water was produced by a Millipore Mili-Q^®^ academic system (Darmstadt, Germany). The secondary effluent (SE) was collected (October, 2017) from Rincón de Caya UWWTP located in Badajoz (Spain), filtered (Whatman Grade 1), and kept frozen until use.

### 3.2. Experimental Procedure and Set-Up

Experiments were carried out in a 0.53 L spherical borosilicate glass reactor with inlet and outlet for the gas and a liquid sampling port. The reactor was placed in the chamber of a solar simulator (Suntest CPS+, Atlas, Linsengericht, Germany) provided with a 1500 W Xe lamp programmed to emit an irradiance of 550 W m^−2^ in the range 300–800 nm. In some experiments, a polyester cut-off filter (Edmun Optics, York, United Kingdom) was used to restrict radiation emission to visible light (λ > 390 nm). In a typical photocatalytic ozonation experiment, the reactor was loaded with 0.5 L of an aqueous solution of PRM (5 mg L^−1^) at natural pH (pH_0_~6) or 0.5 L SE spiked with PRM (5 mg L^−1^ or 100 µg L^−1^). In both cases, 0.125 g of catalyst was added. The suspension was magnetically stirred in the dark for 30 min to reach the adsorption equilibrium. Finally, the Xe lamp was turned on and 20 L h^−1^ of an O_2_-O_3_ mixture containing 10 mg L^−1^ O_3_ (O_3_ mass flow rate 3.3 mg min^−1^) produced by an Anseros Ozomat Com AD-02 generator from O_2_ (purity > 99.5, Linde) was fed to the reactor. At regular intervals, samples were withdrawn from the reactor, filtered (0.45 µM, PVDF Millipore), and analyzed. Total reaction time was 1 and 2 h for the experiments performed in ultrapure water and SE, respectively. This general experimental procedure was adapted to each type of process: no irradiation and/or no O_3_ (only O_2_) and/or no catalyst, etc.

### 3.3. Analytical Methods

Primidone concentration was determined by HPLC-DAD (Hitachi, Elite LaChrom, San Jose, CA, USA) using a Phenomenex C-18 column (3 × 150 mm, 5 μm) as the stationary phase, and 0.6 mL min^−1^ of acetonitrile-acidified water (0.1% phosphoric acid) as mobile phase (20/80 *v*/*v*, isocratic mode). Detection was set at 215 nm. The limit of detection (LOD) of PRM was 100 µg L^−1^ with this method. Low concentration samples (<100 µg L^−1^) PRM were analyzed by HPLC-LC/MS (Agilent 1290 Infinity HPLC coupled to an Agilent 6460 Triple Quadrupole LC/MS) (Santa Clara, CA, USA) using a Zorbax Extend C18 column (3 × 100 mm, 1.8 μm). A gradient elution (flow rate 0.45 mL min^−1^) of water with 0.1% formic acid (phase A) and acetonitrile with 0.1% formic acid (phase B) was applied varying the volume percentage of A solvent from 90% to 0% over 12 min. Using this method, LOD of PRM was 5 µg L^−1^. Identification of PRM transformation products was performed by HPLC-qTOF using an Agilent 6520 accurate mass quadrupole time-of-flight mass spectrometer bearing with electrospray ionization (ESI) source coupled with an Agilent 1260 series LC system (Santa Clara, CA, USA). A Zorbax ECLIPSE PLUS C18 column (4.6 × 100 mm, 3.5 μm) was used as stationary phase and kept at 30 °C. A gradient of Acetonitrile-acidified water (0.1% acetic acid) was used as mobile phase with a constant flow rate of 0.3 mL min^−1^, acetonitrile proportion increases from 5 to 100% of in 35 min and 23 min of equilibration is required for a proper compound separation and elution. The injection volume was 5 µL. The qTOF instrument was operated in the 4 GHz high-resolution mode. Ions are generated using an electrospray ion source Dual ESI. Electrospray conditions were the following: Capillary, 3500 V; nebulizer, 45 psi; drying gas, 10 L min^−1^; gas temperature, 325 °C; skimmer voltage, 65 V; octapoleRFPeak, 750 V; fragmentor, 175 V. The mass axis was calibrated using the mixture provided by the manufacturer over the *m*/*z* 70–3200 range. A sprayer with a reference solution was used as continuous calibration in positive ion using the following reference masses: *m*/*z* 121.0509 and 922.0098. Data were processed using the Agilent Mass Hunter Workstation software (version B.04.00).

Total organic carbon (TOC) was measured using a Shimadzu TOC-VSCH analyser (Kioto, Japan). Short chain organic acids and inorganic anions were determined by a Metrohm 881 Compact Pro ionic chromatograph with chemical suppression equipped with a conductivity detector, using a MetroSep A Supp 7 column (4 × 150 mm, 5 µm) at 45 °C and 0.7 mL min^−1^ of Na_2_CO_3_ from 0.6–14.6 mM in 50 min (10 min post-time for equilibration) as mobile phase. The concentration of ozone in the gas phase (inlet and outlet streams) was monitored by two online analysers (Anseros Ozomat GM-6000) (Tübingen, Germany). Concentration of dissolved ozone in water was determined by the indigo method at 600 nm [59]. Hydrogen peroxide formed was determined by the cobalt/bicarbonate method [60]. For UWWSE characterization, chemical oxygen demand (COD) was measured using Hach Lange LCK 1414 cuvette test kits, N as NH_4_^+^ using a Spectroquant^®^ 114752 test kit from Merck (Darmstadt, Germany), and biochemical oxygen demand (BOD_5_) using an OxiTop® device [61].

The emission spectrum of the Xe lamp was registered with a spectral-radiometer Black Comet C (StellarNet). Using potassium ferrioxalate as chemical actinometer [62] and taking into account the emission spectrum of the lamp, the intensity of UV-Vis (300–800 nm) and Vis (390–800 nm) solar radiation reaching the reaction medium was found to be 8.2 × 10^−5^ Einstein L^−1^ s^−1^ and 7.75 × 10^−5^ Einstein L^−1^ s^−1^, respectively. According to these values, UV solar intensity (300–390 nm) resulted to be 4.6 × 10^−6^ Einstein L^−1^ s^−1^.

Ecotoxicity analyses of PRM in ultrapure water before and after the application of different AOPs were carried out. Acute toxicity tests were conducted with *Daphnia magna* using the commercial test DAPHTOXKIT FTM (Creasel BVBA; Deinze, Belgium) in accordance with testing conditions prescribed by OECD Guideline 202 [63] and using K_2_Cr_2_O_7_ as a reference compound. All the samples were previously diluted 1/8 with the culture medium. Immobility was observed after 15 s of gentle agitation at 24 and 48 h, the latter being the endpoint for effect calculation. In case the percentage of immobilization is lower than 10% it can be considered that the solution does not show acute toxicity to *D. magna*.

## 4. Conclusions

According to the results obtained in this work, ozonation led to total conversion of primidone in less than 30 min regardless of the type of water matrix. When ozone is combined with UV-Vis radiation and/or a photocatalyst a clear positive impact on TOC mineralization is observed. In all cases, HO· radicals resulted to be the main species involved in primidone and TOC removal.

In both, ultrapure water and secondary effluent, the UV-VIS/TiO_2_/O_3_ system led to the highest primidone degradation and TOC mineralization rates, the synergism between ozonation and photocatalysis being more evident at low O_3_ doses. Although the effectiveness of this system in terms of organic nitrogen mineralization was high, it resulted to be lower than for organic carbon. 

Contrary to TiO_2_ P25, when WO_3_ is used as photocatalyst, the presence of dissolved O_3_ as e^−^ acceptor is needed. Because of that, poor mineralization results were obtained when a secondary effluent was treated. In general, the use of WO_3_ did not bring any beneficial effects compared to TiO_2_ P25.

Based on the intermediates identified during ozonation and photocatalytic ozonation of primidone (hydroxyprimidone, phenyl-ethyl-malonamide, 5-ethyldihydropirimidine-4,6(*1H,5H*)-dione, and different carboxylates), a possible degradation pathway is proposed.

For all the systems applied, neither the untreated nor the treated samples (diluted 1/8) were toxic to *D. magna*.

## Figures and Tables

**Table 1:** Main characteristics of the secondary effluent from the MWWTP of Badajoz (Spain).

Parameter	Value	Units
pH	8.2	-
Electrical conductivity	533	µS cm^−1^
COD	47	mg L^−1^ O_2_
BOD5	11	mg L^−1^ O_2_
TOC	14.16	mg L^−1^
DOC	13.8	mg L^−1^
IC	28.8	mg L^−1^
Alkalinity ^(1)^	240	mg L^−1^ CaCO_3_
A_254nm_	0.222	-
SUVA_254_	1.61	L (mg DOC m) ^−1^
F^−^	0.43	mg L^−1^
Cl^−^	78.30	mg L^−1^
NO_3_^−^	22.6	mg L^−1^
NO_2_^−^	0.18	mg L^−1^
SO_4_^=^	52.5	mg L^−1^
PO_4_^3−^	2.6	mg L^−1^

^(1)^ Calculated from IC content.

**Table 2:** Apparent pseudo-fist order rate constants of PRM degradation by different AOPs. Influence of the water matrix.

System	Ultrapure Water k_Obs_, min^−1^ (R^2^)	Secondary Effluent k_Obs_, min^−1^ (R^2^)	Ratio k_Obs(SE)_/k_Obs_
O3	0.321 (0.98)	0.122 (0.99)	0.38
UV-Vis/O_3_	0.429 (0.98)	0.140 (0.99)	0.33
UV-Vis/TiO_2_	0.093 (0.99)	0.011 (0.99)	0.12
UV-Vis/TiO_2_/O_3_	0.380 (0.94)	0.254 (0.98)	0.67
Vis/WO_3_/O_3_	0.224 (0.99)	0.073 (0.98)	0.32

## Supplementary Materials

The following are available online. Figures S1 and S2: Determining ozone-primidone reaction rate constant by absolute method and Figure S3 about changes of hydrogen peroxide concentration with time.
