# Supplementary material for: Ozone-Based Advanced Oxidation Processes for Primidone Removal in Water using Simulated Solar Radiation and TiO2 or WO3 as Photocatalyst
Source: Molecules. 2019 May 3;24(9):1728. doi: 10.3390/molecules24091728 (PMC6539243; doi:10.3390/molecules24091728)
Supplement: Supplementary file 1 [file molecules-24-01728-s001.pdf]

Supplementary information

Ozone based AOPs for primidone removal in water using simulated solar radiation and TiO_2 or WO_3 as photocatalyst

Manuel Figueredo, Eva M. Rodríguez, Manuel Checa and

Fernando J. Beltrán*

Departamento de Ingeniería Química y Química Física. Instituto Universitario de Investigación del Agua, Cambio Climático y Sostenibilidad. Universidad de Extremadura. 06006 Badajoz. Spain.

*corresponding author email: manuelfigueredo@unex.es

Absolute method for the determination of ozone-Primidone reaction rate constant

Experimental procedure: A mixture of primidone ($C_{PRM,0}$ 2.3x10^{-5} M) and tert-butyl alcohol ($C_{TBA,0}$ 2.5x10^{-3} M) in ultrapure water was ozonized ($C_{O3g,inlet}$ = 10 mg L^{-1}; Q_{O2-O3} = 20 L h^{-1}) at pH 2 and 20 ºC, and the evolution with time of PRM and dissolved ozone concentrations analyzed. At these conditions, degradation of PRM takes place only through its direct reaction with molecular ozone.

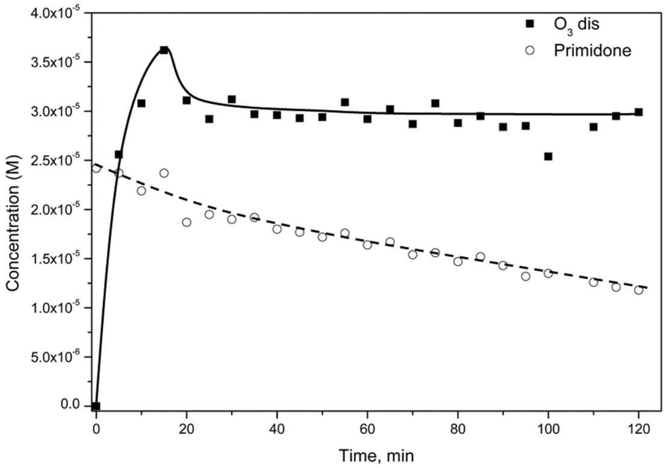

Figure S1: Evolution of PRM and dissolved O_3 concentrations with time during the ozonation of PRM in presence of TBA (two replicates). Experimental conditions: $C_{PRM,0}$ = 2x10^{-5} M; $C_{TBA,0}$ = 2.3x10^{-3} M; pH = 2; T = 20 ºC; $C_{O3g,inlet}$ = 10 mg L^{-1}; Q_{O2-O3} = L h^{-1}.

Calculus: In the semibatch perfectly mixed reactor used in this work, at the conditions tested and assuming a second-order kinetics for the reaction between ozone and PRM, PRM mass balance can be expressed as follows:

$$-\frac{dC_{PRM}}{dt} = k_D C_{PRM} C_{O3} \qquad \text{(S1)}$$

As observed in Figure S1 dissolved ozone concentration in solution (C_{O3}) reached a stationary state (C_{O3ss}) after 25 minutes (t_{ss}). Hence, for higher reaction times a pseudo first order kinetic model can be assumed:

$$-\frac{dC_{PRM}}{dt} = k'_D C_{PRM} \qquad \text{(S2)}$$

where $k'_D = k_D C_{O3ss}$, being $C_{O3ss} = 2.9\text{x}10^{-5}$ M (see Fig S1).

The integration of equation (S2) leads to equation (S3):

$$\ln\frac{C_{PRMss}}{C_{PRM}} = k'_D\left(t - t_{ss}\right) \qquad \text{(S3)}$$

where C_{PRMss} is the concentration of PRM at t_{ss}.

Thus, k'_D value can be obtained by fitting experimental data to eq. (S3) as shown in Figure S2. From least squares analysis of the straight line shown in this figure k'_D resulted to be $9.02\cdot10^{-5}$ s^{-1} (R^2=0.97). Therefore, the second-order rate constant of the reaction between PRM and O_3 is $k_D = 3.08$ $M^{-1}s^{-1}$.

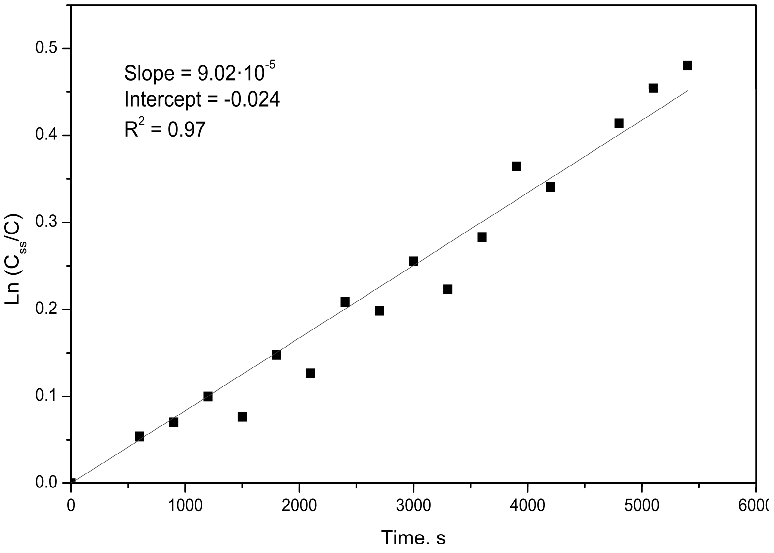

[illegible]

Once the rate constant was obtained the slow kinetic regime of ozone absorption was verified determining the Hatta number by equation S4 [1]). In eq. S4 D_{O3} is the diffusivity of ozone in water ($1.76\text{x}10^{-9}$ m^2 s^{-1}; [2]) and k_L the individual liquid-side mass transfer coefficient calculated as $3.74\text{x}10^{-4}$ m s^{-1} from Calderbank's equation [3]. For slow kinetic

regime Ha is < 0.3 [1].

$$Ha = \frac{\sqrt{k_D\ C_{PRM}\ D_{O3}}}{k_L} \qquad (S4)$$

According to S4 from the estimated k_D and the experimental C_{PRM} data, Ha resulted to be < 0.3 which validates the value of k_D obtained.

Concentration of hydrogen peroxide during photocatalytic oxidation processes studied in this work

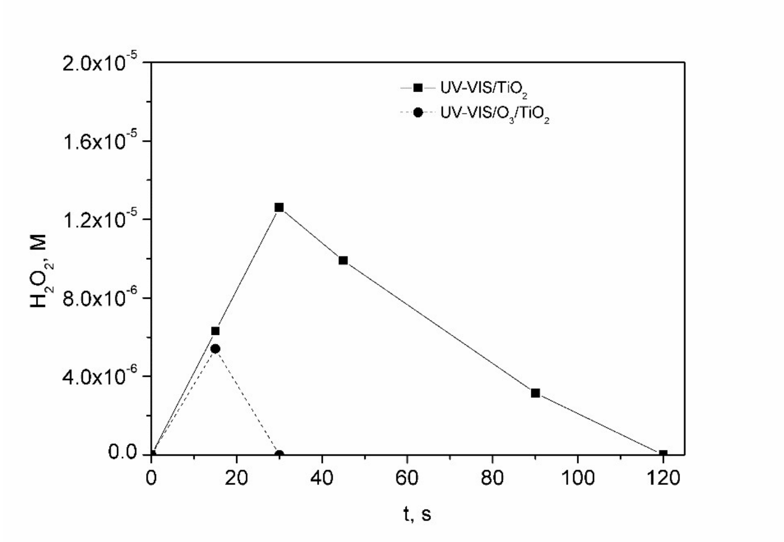

Figure S3. Changes of hydrogen peroxide concentration with time during PRM oxidation with UV-Vis/TiO_2 and UV-Vis/TiO_2/O_3 systems. Experimental conditions: Ultrapure water; $[PRM]_0$ = 5 mg L^{-1}; pH_0 ~ 6 (natural pH); [TiO2] = 0.25 g L^{-1}; $Q_{m,O3}$ = 3.3 mg min^{-1} ; T = 20-30 ºC; IUV-Vis = 8.2 x 10^{-5} Einstein L^{-1} s^{-1}.

References

[1] J.C. Charpentier, Mass-transfer rates in gas-liquid absorbers and reactors, Adv. Chem. Eng. 11 (1981) 1–133. doi:10.1016/S0065-2377(08)60025-3.

[2] P.N. Johnson, R.A. Davis, Diffusivity of ozone in water, J. Chem. Eng. Data. 41 (1996) 1485–1487. doi:10.1021/je9602125.

[3] G.F. Froment, K.B. Bischoff, J. De Wilde, Chemical Reactor Analysis and Design, 3rd edition, Jonh Wiley & Sons, Inc., USA, 2011.
